# Does Income Diversification Benefit the Sustainable Development of Chinese Listed Banks? Analysis Based on Entropy and the Herfindahl–Hirschman Index

**DOI:** 10.3390/e20040255

**Published:** 2018-04-06

**Authors:** Huichen Jiang, Liyan Han

**Affiliations:** School of Economics and Management, Beihang University, Beijing 100191, China

**Keywords:** commercial banks, Herfindahl–Hirschman index (HHI), entropy index (ENTI), profitability, risk, income diversification, threshold effect

## Abstract

We collected data pertaining to Chinese listed commercial banks from 2008 to 2016 and found that the competition between banks is becoming increasingly fierce. Commercial banks have actively carried out diversification strategies for greater returns, and the financial reports show that profits are increasingly coming from the non-interest income benefits of diversification strategies. However, diversification comes with risk. We built a panel threshold model and investigated the effect of income diversification on a bank’s profitability and risk. Diversification was first measured by the Herfindahl–Hirschman index (HHI), and the results show that there is a nonlinear relationship between diversification and profitability or risk does exist. We introduced an interesting index based on the entropy to test the robustness of our model and found that a threshold effect exists in both our models, which is statistically significant. We believe the combination of the entropy index (ENTI) and the HHI enables more efficient study of the relationship between diversification and profitability or risk more efficiently. Bankers and their customers have increasingly been interested in income diversification, and they value risk as well. We suggest that banks of different sizes should adopt the corresponding diversification strategy to achieve sustainable development.

## 1. Introduction

### 1.1. Background

Since 2006, the reform of China’s banking industry has taken a new and important step. The “Big Five” banks, the Bank of China Limited (BOC), Industrial and Commercial Bank of China Limited (ICBC), China Construction Bank Corp. (CCB), Agricultural Bank of China Limited (ABC), and Bank of Communications Co., Ltd. (BOCOM), have successfully completed shareholding system reform and are listed on the Chinese A-share stock market. At the same time, the reform of interest rate marketization (IRM) has been steadily promoted. Zhou Xiaochuan, China’s central bank chief, pointed out that IRM enlarged the banks’ pricing power and the pricing power on loans and deposits. Moreover, the reform will help the market play a role in allocating resources and reflect the independent pricing power of financial institutions on their products and services. In addition, customers can choose from similar financial products at different prices, so financial institutions will be able to provide a variety of financial products and services and offer different prices according to the customer’s risk [1]. Additionally, in recent years, the Chinese government has emphasized the importance of promoting IRM reform in reports on the work of the government. 

**Figure 1:**
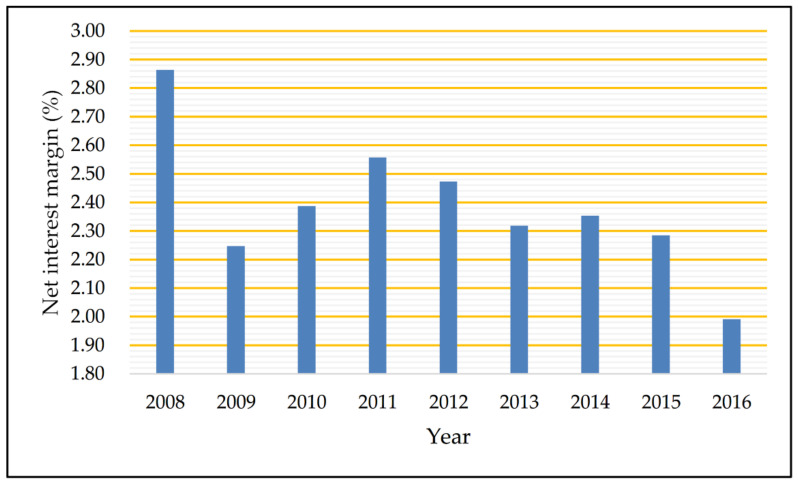
The net interest margin of listed commercial banks.

**Figure 2:**
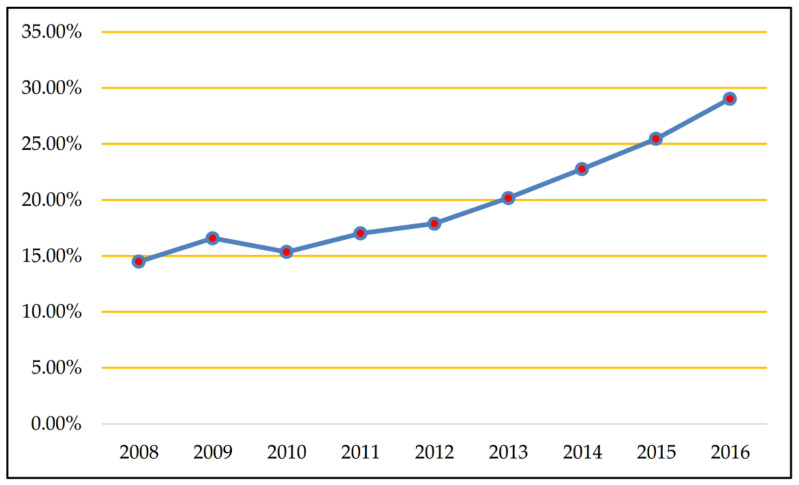
The percentage of non-interest income in operating income.

The outbreak of the global financial crisis in 2008 had a substantial and far-reaching impact on China’s banking industry. Against the background of the global financial crisis, with the continuous reforms and innovations of China’s financial system, interbank competition has intensified. According to our calculation, the net interest margin has been volatile over the years (see Figure 1), which means the traditional operating strategy based on interest income should be changed. Therefore, there is an urgent demand for Chinese commercial banks to transform and upgrade main businesses, and many banks have adopted the strategy of income diversification, by providing more abundant financial products and services (non-interest business) to broaden their income channels. As shown in Figure 2, non-interest income has increasingly contributed to operating income from 2008 to 2016. 

### 1.2. The Income Diversification of Banks

Income diversification has become an important trend in the development of modern banking. Most early studies were focused on the income diversification strategies of the European and American banking industries and described the potential benefits of income diversification from a variety of perspectives. First, by engaging in a wider range of financial activities, diversification can broaden revenue channels and cultivate new growth points. Second, banks can achieve operational synergies in providing a variety of financial products and services and enhance profitability with the economies of scale. In addition, since the non-interest business of a bank is irrelevant or not completely relevant to the interest business (which was believed to be more highly related to the economy), the diversification of income structures can reduce the volatility of the bank’s income and stabilize it. Furthermore, by making full use of the customer information accumulated in traditional businesses, universal banks that adopt income diversification incur lower costs than specialized banks engaged only in traditional banking (see [2,3,4,5]). Moreover, some scholars believe that universal banks achieve well-diversified incomes and face lower risks than specialized banks. According to statistics from the Great Depression in the United States, most of the bankrupt banks were specialized, engaging only in a single business. To summarize, income diversification makes banks operate more efficiently by reducing cost, lowering risk, and enhancing profitability. Additionally, it can contribute to financial stability and economic development (see [6,7]). 

With the development of non-interest business all over the world, research can deepen our understanding of the effect of diversification on banks’ profitability and risk. Studies have increasingly provided empirical evidence showing that diversification can benefit banks. Pennathur et al. [8] concluded that diversification benefited the public-sector banks of India by reducing default risk during the period 2001–2009. Shim [9] found that diversification reduced the likelihood of insolvency risk based on data of a USA bank holding company from 1992 to 2011, as the diversified income portfolio lowered the volatility of income. Other studies have found that income diversification may increase risk and reduce income and stability, theoretically and empirically. Lepetit et al. [10] found that there was a positive relationship between fee-based activities (commission income, which is a type of non-interest income) and solvency risk based on the data of European banks from 1996 to 2002. Hayden et al. [11] showed that, for most German banks, a higher level of diversification may result in a lower return, by using data of the loan portfolios of banks during the 1996–2002 period. Using data pertaining to banking in the USA from 1997 to 2002, Stiroh [12] analyzed the influence of income diversification on bank performance, and found that decreased interest income volatility contributed to income stability, while non-interest income was more volatile than interest income. Additionally, components of non-interest income, such as service charges and fees, are highly correlated with interest income. According to income structure statistics on the European banking industry from 1994 to 2002, Wang pointed out that, although the contribution of non-interest income to operating income increased significantly during the period, the growth of non-interest income to some extent compensated for the decline in interest income. However, the data show that, in 1995 and 1997, the rise in non-interest income was accompanied by a certain degree of increased operating costs. In other words, the impact of non-interest income on the profitability of banks is, on the whole, uncertain [13]. Additionally, Mercieca et al. [14] found that there was no direct diversification benefit for small European banks from 1997 to 2003, as they lacked experience and expertise on new types of business. Some researchers explored the heterogeneous effect of diversification, while some found that this effect does not exist. According to Baele et al., the diversification gains and costs are not significant for small and large banks in the European Union [15]. Hidayat et al. [16] studied the relationship between risk and diversification in Indonesian banks, and the results showed that the effect highly depends on the scale of the bank.

In sum, most research is currently focused on banks in the U.S. or in E.U. member countries, and the financial system and level of financial development in China are different from these countries. Therefore, whether or not income diversification is advantageous to Chinese banks is of great importance. In addition, there are no unanimous conclusions regarding the relationship between a bank’s profitability and risk. Some people believe income diversification is beneficial for banks by improving profitability and income stability, while others argue that diversification may increase risk and income volatility. Moreover, it is worth noting that some research found that diversification may have a heterogeneous impact on banks. Therefore, we decided to use the panel threshold model to check whether the effects of diversification on profitability and risk are different among banks.

### 1.3. The Diversified Use of Entropy

Entropy is an interesting method, which was originally created and introduced in physics in the middle of the nineteenth century. It can be said that the entropy and its application formed the basis of statistical mechanics for understanding the conversion from heat to motive force. The idea of entropy is simple and easy to understand and has been developed by scientists and widely applied beyond the area of physics (see Scarfone [17], Martyushev and Seleznev [18], Tsallis and Souza [19], Pressé et al. [20]). According to Gulko [21], the first introduction of entropy in economic areas could date back to the 1960s. The entropy has been considered as a useful statistic and used in financial areas in the 1990s, Stutzer [22] and Avellaneda [23] made substantial and significant contributions to the application of entropy in finance. 

Nowadays, the concept of entropy has been applied widespread in the areas of economic and financial studies. 

The concept of entropy could be used in the pricing of financial products. By using the minimization of relative entropy, Stutzer [24] provided a simple way to derive the Black–Scholes option pricing model. Additionally, Kitamura et al. [25] pointed out that the entropy-based approach performs better than the traditional linear approach if non-normalities of observations exist. Inspired by Buchen et al. [26], Brody et al. [27], based on Rényi entropy, designed an entropic pricing method that required fewer parameters than the traditional approach.

The entropic method is also used in analyzing financial risk and financial crises. In the analysis of interbank contagion, the maximum entropy approach has become predominant (see [28]). Based on the concept of directional entropy, Bowden [29] enhanced a favored financial risk management tool called value at risk (VaR), which can measure tail risk. Pele et al. [30] proved that the entropy of the distribution function of intraday returns can predict classical measures of market risk, including the VaR, and had more informational content than the traditional ones. Yang and Qiu [31] believed that the introduction of entropy can contribute to decision-making. Based on Tsallis [32], Gençay and Gradojevic [33,34] introduced and developed an entropic approach as a measure of market expectations, and their further research [35] showed the application of Tsallis entropy and approximate entropy in finance to be a good predictor of the financial crises in 1987 and 2008. Boyarchenko [36] used relative entropy to measure the implied amount of ambiguity investors face, based on Hansen and Sargent [37]. 

Additionally, the entropy can be applied in financial time series analysis. Bekiros [38] designed a shift-invariant discrete wavelet transform (SIDWT), which uses the entropy-based criterion of obtaining the optimal depth of wavelet decomposition. The new methodology is superior to the traditional subjective approach and can be used in financial time series analysis. Dimpfl [39] discussed how transfer entropy can be applied to analyzing the information flows among financial markets. Compared with the Granger causality, Zaremba [40] pointed out that the transfer entropy can identity the nonlinear dependence better. Besides, Geman et al. [41] filled the gap of traditional financial literatures by analyzing the multi-dimensional density of the returns of assets with entropic framework.

As mentioned above, the aim of the research is to identity the impacts of income diversification on the return and risk of banks. Therefore, the measure of diversification is crucial for our research. As prior researches proved that, the Herfindahl–Hirschman Index (HHI) can be a good measure of diversification [42], while Tabak et al. [43] showed that entropic index can also be used as a measure of diversification. Therefore, we follow the traditional research and use the HHI to measure the diversification of banks and the entropy index (ENTI) is used for checking the robustness of our results.

The rest of the paper is organized as follows: Section 2 outlines the methodology of the panel threshold model (PTM), our theoretical assumptions, and the selection of variables and data sources. In Section 3, we describe the data and examine whether the effects of diversification on profitability, credit risk and the overall solvency risk of banks differ with the PTM. The findings and conclusions are summarized in Section 4.

## 2. Materials and Methods

### 2.1. Panel Threshold Model

According to the literature review in Section 1, the relationship between a bank’s profitability (or risk) and income diversification is probably nonlinear. That is to say, the effects of diversification on different banks vary. In order to determine whether a nonlinear relationship exists, the PTM, which was first designed by Hansen [44], was considered suitable for our research. 

According to [44,45,46,47], the single-threshold model can be written as follows:

(1) $$y_{it}=\mu_{i}+\beta_{1}DIV_{it}I\left(q_{it}\leq r\right)+\beta_{2}DIV_{it}I\left(q_{it}>r\right)+\varepsilon_{it}$$

where $y_{it}$ refers to the dependent variable, namely the bank’s profitability or its risk; $DIV_{it}$ refers to the explanatory variable, namely the level of bank’s income diversification; $\mu_{i}$ and $\varepsilon_{it}$ are the individual-specific effect and the stochastic disturbance, respectively; $q_{it}$ is the threshold variable; r is the threshold parameter to be estimated. I(A) is a characteristic function as 1 if A occurs and 0 if A does not occur. Clearly, Equation (1) is divided into 2 regimes with the threshold parameter.

In order to eliminate the unobserved individual-specific effect, first, for each i, Equation (1) is averaged over time as follows:

(2) $$\bar{y_{i}}=\mu_{i}+\beta_{1}\bar{DIV_{i}}I\left(q_{it}\leq r\right)+\beta_{2}\bar{DIV_{i}}I\left(q_{it}>r\right)+\bar{\varepsilon_{i}}.$$

For simplicity, we define

(3) $$\beta =\left(\begin{array}{c} \beta_{1}\\ \beta_{2} \end{array}\right)$$

(4) $$DIV_{it}\left(r\right)=\left(\begin{array}{c} DIV_{it}I\left(q_{it}\leq r\right)\\ DIV_{it}I\left(q_{it}>r\right) \end{array}\right).$$

Equations (1) and (2) can then be expressed as follows:

(5) $$y_{it}=\mu_{i}+\beta^{\prime }DIV_{it}\left(r\right)+\varepsilon_{it}$$

(6) $$\bar{y_{i}}=\mu_{i}+\beta^{\prime }\bar{DIV_{it}\left(r\right)}+\bar{\varepsilon_{i}}.$$

Second, we subtract Equation (6) from Equation (5), and $DIV_{it}^{*}$ and $\varepsilon_{it}^{*}$ are defined as follows:

(7) $$y_{it}^{*}=y_{it}-\bar{y_{i}}$$

(8) $$DIV_{it}^{*}=\beta^{\prime }\left[DIV_{it}\left(r\right)-\bar{DIV_{it}\left(r\right)}\right]$$

(9) $$\varepsilon_{it}^{*}=(\varepsilon_{it}-\bar{\varepsilon_{i}}).$$

We then yield the time-demeaned form as follows:

(10) $$y_{it}^{*}=\beta^{\prime }\left[DIV_{it}\left(r\right)-\bar{DIV_{it}\left(r\right)}\right]+(\varepsilon_{it}-\bar{\varepsilon_{i}})=\beta^{\prime }DIV_{it}^{*}+\varepsilon_{it}^{*}.$$

With the threshold parameter (r), we can estimate the coefficient ($\hat{\beta }$) by using the ordinary least squares (OLS) method and calculate the sum of squared residuals (SSR), which is denoted as $S_{1}\left(r\right)$. The threshold parameter can be estimated by minimizing $S_{1}\left(r\right),$ and we can estimate $\hat{\beta }$ given the estimator of threshold parameter ($\hat{r})$.

After estimating the parameters, we need to check whether the threshold effect is statistically significant. The null hypothesis and alternative hypothesis are as follows:

(11) $$H_{0}:\beta_{1}=\beta_{2}$$

(12) $$H_{1}:\beta_{1}\neq \beta_{2}.$$

If $\beta_{1}=\beta_{2}$, there is no threshold effect, Equation (5) can be written as follows, and we can derive the time-demeaned form in Equation (14), yield the estimator of coefficient, and calculate SSR ($S_{0}$) under the assumption of $\beta_{1}=\beta_{2}$:

(13) $$y_{it}=\mu_{i}+\beta_{1}{}^{\prime }DIV_{it}^{*}+\varepsilon_{it}$$

(14) $$y_{it}^{*}=\beta_{1}{}^{\prime }DIV_{it}^{*}+\varepsilon_{it}^{*}$$

(15) $$S_{0}=\tilde{\varepsilon_{it}^{*}{}^{\prime }}\varepsilon_{it}^{*}.$$

Based on [44], the likelihood ratio (LR) test statistics of $H_{0}$ are as follows. We can use the bootstrap method introduced by [45] to attain the asymptotic distribution of the following test and calculate the *p*-value:

(16) $$F=\frac{S_{0}-S_{1}\left(\hat{r}\right)}{\hat{\sigma^{2}}}$$

where the residual variance $\hat{\sigma^{2}}=\frac{S_{1}\left(\hat{r}\right)}{n\left(T-1\right)}$, and n and T refer to the number of individuals and time periods, respectively.

Besides, we also need to check whether the estimated threshold parameter ($\hat{r}$) is equal to the true value ($r_{0}$), namely $\hat{r}=r_{0}$. The corresponding statistic is shown in Equation (17). Meanwhile, the distribution of LR statistic is non-standard. Therefore, a nonrejection region method for the test was designed by [46]. We cannot reject the null hypothesis that $\hat{r}=r_{0}$ at a significance level ∝ if LR(r) is less than $-2\log\left(1-\sqrt{1-\propto }\right),$ which suggests that the threshold parameter is equal to the true value. Note that ∝ and 1−∝ represent the significance level and the confidence level, respectively.

(17) $$\mathrm{LR}\left(r\right)=\frac{S_{1}\left(r\right)-S_{1}\left(\hat{r}\right)}{\hat{\sigma^{2}}}.$$

Similarly, we can define a double-threshold model with two threshold parameters dividing the equation into 3 regimes and build the statistics for handling the multiple-threshold situation as above. Note that we need to derive the first threshold and obtain the second threshold by taking the first threshold as given, besides, the first threshold should be reestimated by taking the second threshold as given as the second threshold is asymptotically efficient while the first threshold is not. For a detailed procedure of a multi-threshold model, see Hansen [44].

### 2.2. Variable Selection and Data Sources

In this paper, we are interested in the relationship between income diversification and a bank’s profitability and risk. Therefore, considering the available data and prior studies (see [43,48,49,50,51]), three variables were selected as dependent variables: the risk-adjusted return on assets (SHROA), the non-performing loan ratio (NPLR), and the Z-score.

The SHROA, which represents the bank’s return, is defined as follows:

(18) $$\mathrm{SHROA}=\frac{\mathrm{ROA}}{\sigma_{\mathrm{ROA}}}$$

where ROA and $\sigma_{\mathrm{ROA}}$ refer to the return on assets (ROA) and the standard deviation of ROA during the sample period.

The non-performing loan ratio (NPLR), which reflects the bank’s credit risk, is defined as follows:

(19) $$\mathrm{NPLR}=\frac{\mathrm{Non}-\mathrm{performing}\;\mathrm{Loans}}{\mathrm{Total}\;\mathrm{loans}}.$$

The Z-score, which represents the overall insolvency risk (the inverse of Z-score can be seen as the measure of the bank’s financial stability), is defined as follows:

(20) $$Z-\mathrm{score}=\frac{\sigma_{\mathrm{ROA}}}{\mathrm{ROA}+\left(\frac{\mathrm{Equity}}{\mathrm{Total}\;\mathrm{assets}}\right)}.$$

The diversification index based on the concept of the Herfindahl–Hirschman index (HHI), denoted “DIV,” is defined as follows:

(21) $$\mathrm{DIV}=1-{\mathrm{NIIS}}^{2}-{\mathrm{NNIS}}^{2}$$

where NIIS and NNIS refer, respectively, to the ratio of net interest income to total operating income (i.e., the share of net interest income) and the ratio of net non-interest income to total operating income (i.e., the share of net non-interest income). This has been widely applied in related studies and is used in our baseline model.

Jacquemin and Berry [52] and Ceptureanu et al. [53] used the entropy index (ENTI) as a measure of diversification. The diversification index based on the ENTI, denoted “DEV” and used for the robustness test, is defined as follows:

(22) $$\mathrm{DEV}=\mathrm{NIIS}\times \ln\left(1/\mathrm{NIIS}\right)+\mathrm{NNIS}\times \ln\left(\frac{1}{\mathrm{NNIS}}\right).$$

Note that the DIV and DEV reach their maximums when the share of net-interest income is equal to the share of non-interest income; otherwise, with an increase in the share of net-interest income or non-interest income, the bank will be specialized, and the diversification index decreases.

In addition, the bank’s size, equity ratio, and loan-to-deposit ratio are included to control the other factors that may affect its return and risk. The bank’s size (ast) is measured by the natural logarithm of its total assets. The equity ratio (eta) is defined as the ratio of equity and total assets. The loan-to-deposit ratio (ltd) is defined as the ratio between total loans and total deposits. We manually collected data from the publicly disclosed financial reports of the commercial banks listed on the A-share stock market, and the monetary values of the variables are shown in renminbi (RMB).

## 3. Results

### 3.1. Sample Description

Based on the industry classification of listed firms, we found that there were 24 commercial banks listed on the Chinese A-share stock market as of 31 December 2016. Considering the availability and continuity of data, 16 banks were selected as our sample. We collected data of Chinese listed commercial banks from 2008 to 2016, and found that the competition between banks is becoming increasingly fierce. Commercial banks have actively carried out diversification strategies for greater returns, and the financial reports show that profits increasingly come from non-interest income, a benefit from the diversification strategy. However, diversification comes with risk.

Our sample comprises five state-owned commercial banks: Industrial and Commercial Bank of China Limited (ICBC), China Construction Bank (CCB), Bank of Communications (BOCOM), Agricultural Bank of China (ABC), and Bank of China (BOC); eight joint-stock commercial banks: China Everbright Bank (CEB), Hua Xia Bank (HXB), China Minsheng Banking (CMBC), Ping An Bank (PAB), Shanghai Pudong Development Bank (SPDB), Industrial Bank (IB), China Merchants Bank (CMB), and China CITIC Bank (CITICB); and three city commercial banks: Bank of Nanjing (NJCB), Bank of Ningbo (NBCB), and Bank of Beijing (BJCB). According to data released by the China Banking Regulatory Commission (CBRC) [54], the total assets of the Chinese banking industry reached 181.7 trillion RMB in 2016, and the listed commercial banks in the sample accounted for 73.93% of the total assets, which shows that our sample is sufficiently representative of the entire Chinese banking industry. Table 1 shows the full names and abbreviations of the 16 banks.

Table 2 and Table 3 show descriptive statistics of dependent variables, independent variables, and controlled variables.

### 3.2. Threshold Test

In this section, we test whether a nonlinear relationship between income diversification and a bank’s profitability, credit risk, and the overall insolvency risk exists. Furthermore, if the threshold exists, we examine whether the single-threshold, the double-threshold, or the triple-threshold model is suitable for analyzing the effect of income diversification. 

Table 4, Table 5 and Table 6 show the F-statistics, bootstrap *p*-value, and critical value for the 1%, 5%, and 10% significance levels, respectively, of the threshold effect test when the dependent variable reflects profitability (SHROA), credit risk (NPLR), or overall solvency risk (Z-score). The bootstrap procedure was repeated 300 times for both models. Note that the F-statistics in the following tables are under the null hypothesis of no threshold, at most one threshold, and at most two thresholds, respectively. In Table 4, the F-statistics of single-threshold, double-threshold, and triple-threshold are 22.680, 15.310, and 14.310, respectively. Only the F-statistics of single-threshold is significant at 5% significance level, showing that a nonlinear relationship between profitability and income diversification does exist, and there is one threshold. As shown in Table 5, the reported F-statistics and calculated *p*-value confirm that there is one threshold for credit risk and income diversification. Similarly, Table 6 shows that the F-statistics assessing the null hypothesis of no threshold, at most one threshold, and at most two thresholds are respectively 39.330, 17.150, and 14.960, suggesting that there is one threshold for overall solvency risk and income diversification.

**Figure 3:**
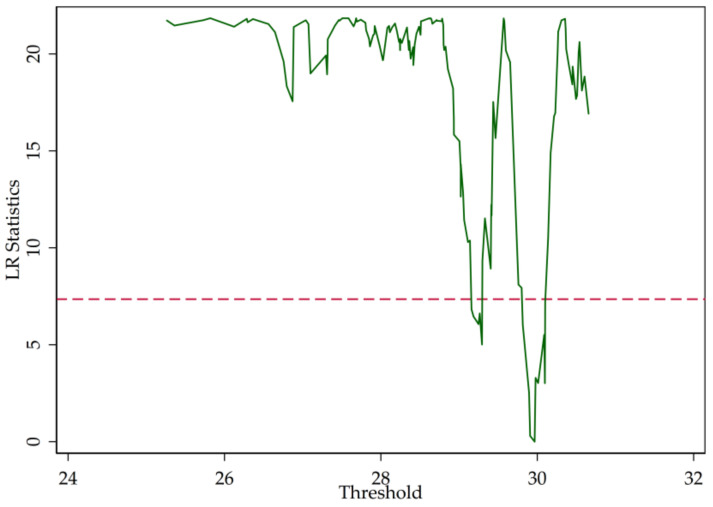
Likelihood ratio (LR) statistics (SHROA).

**Figure 4:**
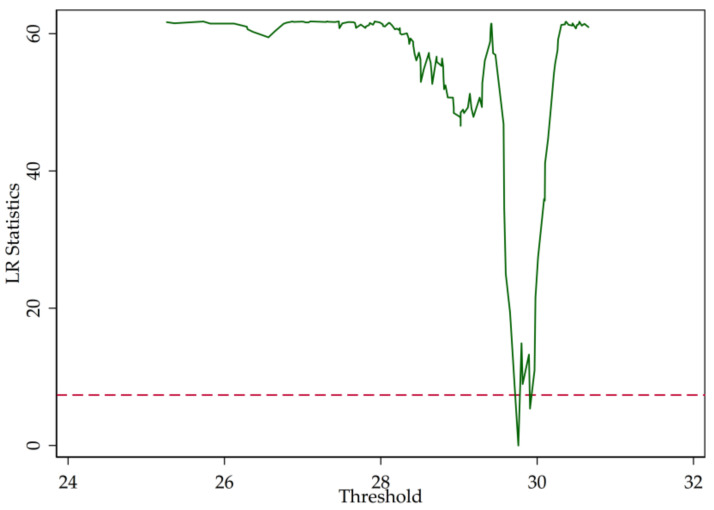
LR statistics (NPLR).

**Figure 5:**
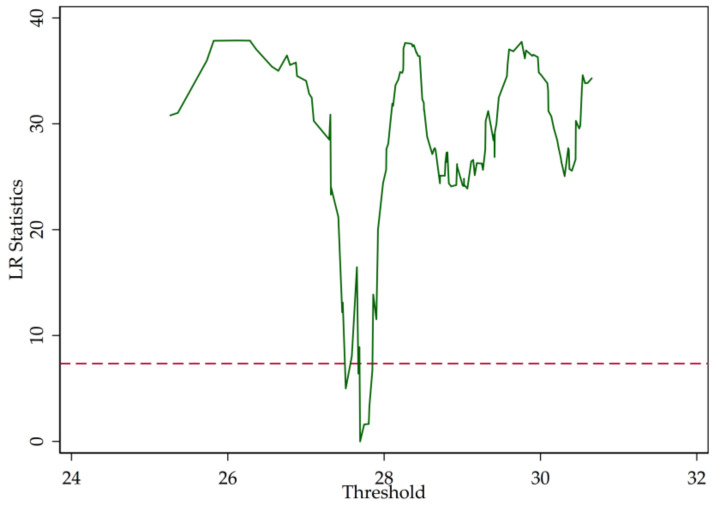
LR statistics (Z-score).

In Section 2, we point out that the LR statistics are the function of the threshold parameter (r). Figure 3, Figure 4 and Figure 5 illustrate the LR statistics of single thresholds of different models. Note that the dashed line represents the critical value at the 95% confidence level. The threshold parameter is derived by letting the LR statistics equal zero.

### 3.3. Estimation Results

The estimations of equations are listed in Table 7. Note that ***, **, and * indicate statistical significance at the 1%, 5%, and 10% levels, respectively; DIV_1 and DIV_2 represent the coefficient of income diversification when the bank size is smaller and larger than the threshold parameter, respectively; N and R^2^ refer to the number of observations in the sample and the statistics indicator, which reflects the goodness of fit. Based on our calculation, the regression of a bank’s profitability on diversification (Column 1) shows that, when the bank size is smaller than the threshold value of 10.33739 trillion RMB, income diversification negatively influences the return, measured by risk-adjusted ROA, and the influence is significant at the 10% level. When the size exceeds the threshold value, the influence is positive but not statistically significant. Furthermore, for the controlled variables, we find that bank size negatively affects the equity ratio and has a positive relationship with profitability, which implies that small banks may perform better than large banks. Additionally, the results show that there is a positive relationship between equity ratio and profitability, significant at the 1% level, which shows that banks with a higher equity ratio are likely to earn more profit. Additionally, banks with a higher loan-to-deposit ratio tend to have lower returns. 

Column 2 shows the estimation results of the regression of diversification on credit risk, measured by the non-performing loan ratio. Based on the calculation and Figure 4, the threshold parameter is 8.403169 trillion RMB. According to Table 7, if the bank size is smaller than the threshold parameter, income diversification increases the non-performing loan ratio, which increases credit risk. However, if the bank is larger than the threshold parameter, income diversification reduces the non-performing loan ratio and the bank has higher-quality loans. The result also shows that the relationship between bank size and the non-performing loan ratio is negative and significant at the 5% level, which may be due to the fact that large banks tend to have superior risk management techniques and abundant human resources, and can therefore efficiently engage in the non-interest income business and enjoy the benefits of diversification. Additionally, the results show that, with rising loan-to-deposit ratio, credit risk grows.

In Column 3 of Table 7, we show the estimation of the regression of profitability on overall solvency risk measured by Z-score. The calculation shows that the threshold parameter is 1.0639 trillion RMB. Based on Table 7, we find that, regardless of the size of a bank, income diversification positively influences overall solvency risk. Moreover, if the bank size is less than 1.063901 trillion RMB, the increased solvency risk is greater than it is for larger banks. In addition, the results show that an increased bank size tends to reduce solvency risk. Additionally, there is a negative relationship between equity ratio and solvency risk, which suggests that, if the bank reduced the equity ratio and raised the proportion of debt to assets, the solvency risk would likely rise, significant at the 1% level. Additionally, there is a positive relationship between the loan-to-deposit ratio and the bank’s solvency risk, significant at the 5% level, which shows that a larger ratio results in increased solvency risk.

### 3.4. Robustness Analysis

In the above section, we analyzed the relationship between income diversification and the bank’s profitability or risk, with the level of diversification measured by the DIV index. We conducted a robustness check, and the DEV index is used to measure diversification level in this section.

The threshold effect tests are listed in the following tables. According to Table 8, Table 9 and Table 10, the number of thresholds is one for different models, which is consistent with the above section. Thus, the results confirm the existence of a nonlinear relationship.

**Figure 6:**
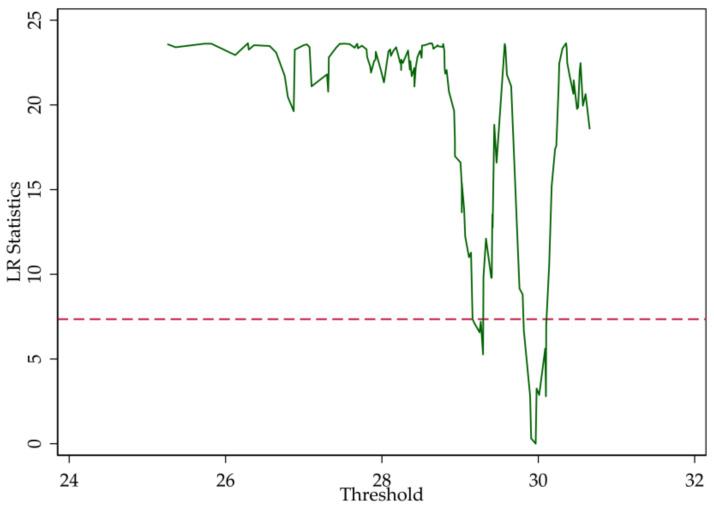
LR statistics (SHROA).

**Figure 7:**
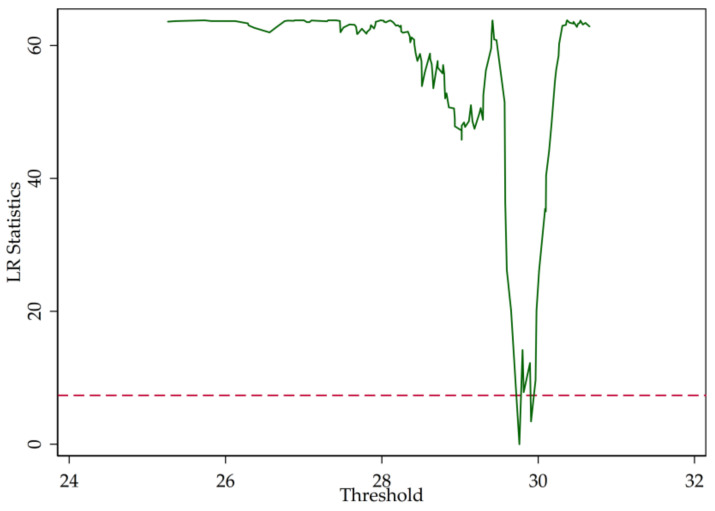
LR statistics (NPLR).

**Figure 8:**
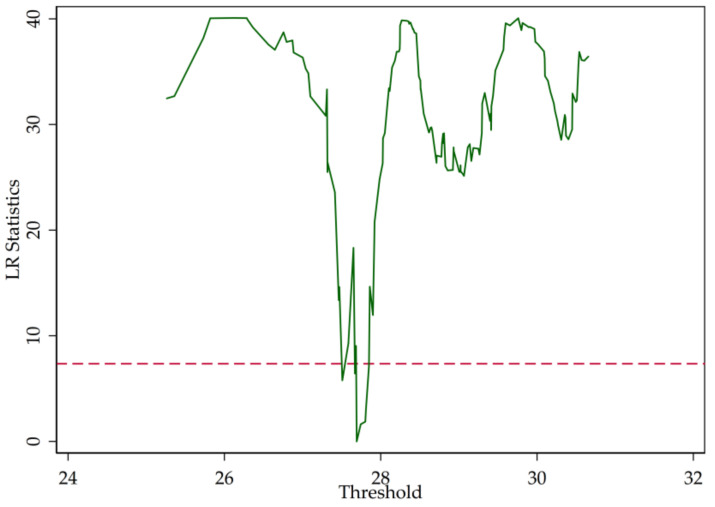
LR statistics (Z-score).

Graphical representations of LR statistics of the models are shown in Figure 6, Figure 7 and Figure 8. Similarly, if we let the LR statistics equal zero, we can derive the threshold parameter. By calculation, we find that the threshold parameters of different models are robust.

Additionally, we estimate Model 1, Model 2, and Model 3 by using the DEV index as a measure of income diversification under the assumption of the existence of one threshold. The results are shown in Table 11. Note that DEV_1 and DEV_2 refer to the coefficient of diversification when bank size is smaller or larger than the threshold parameter, respectively. The results show that there is only a slight difference between the coefficients for the same models with different measures of diversification. Furthermore, the directions of the coefficients do not change, which indicates that our results are robust.

**Figure A1:**
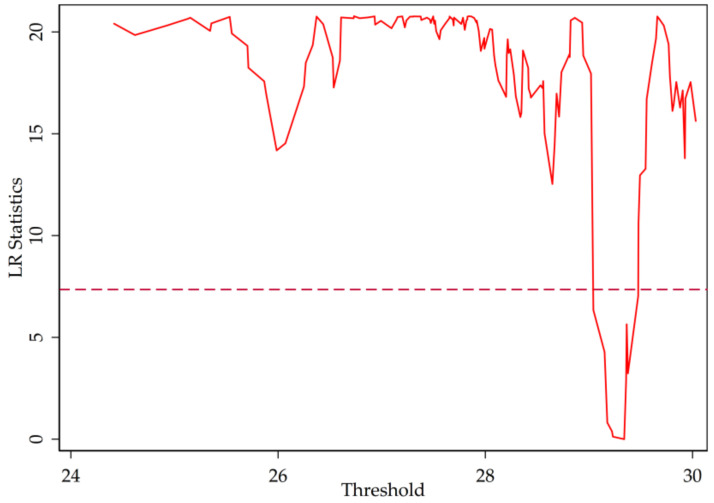
LR statistics (SHROA).

**Figure A2:**
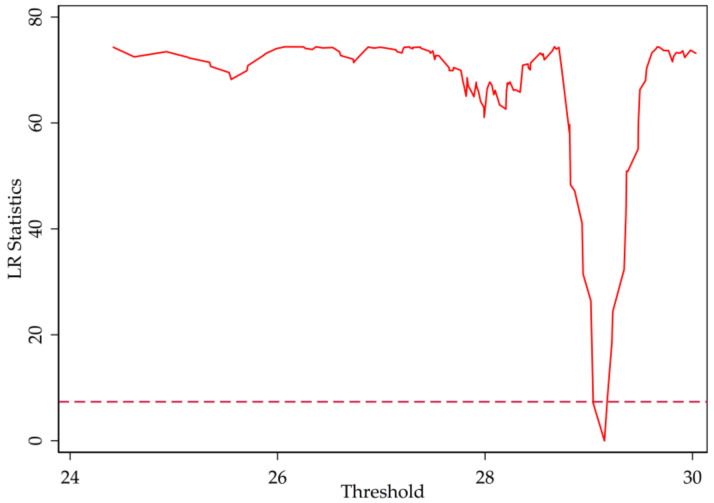
LR statistics (NPLR).

**Figure A3:**
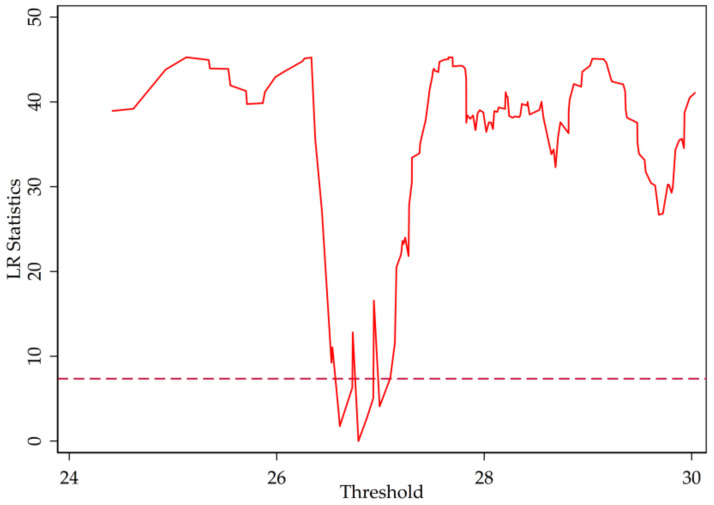
LR statistics (Z-score).

**Figure A4:**
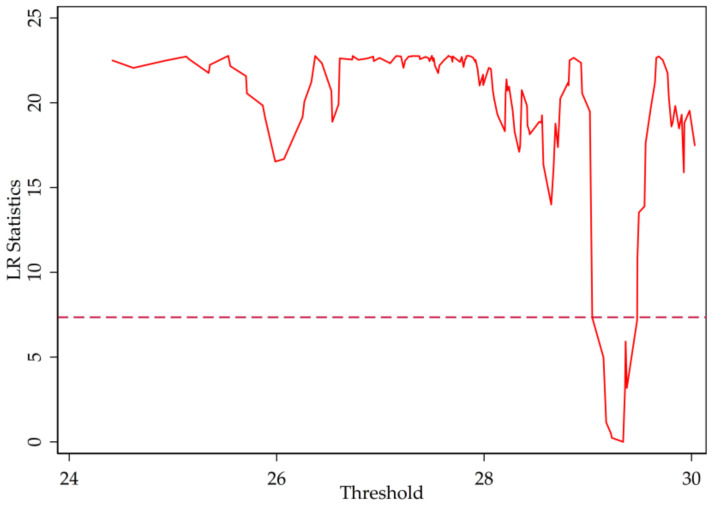
LR statistics (SHROA).

**Figure A5:**
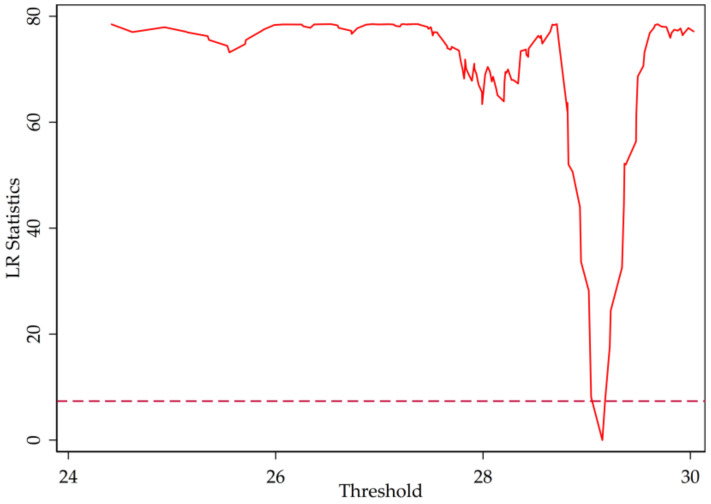
LR statistics (NPLR).

**Figure A6:**
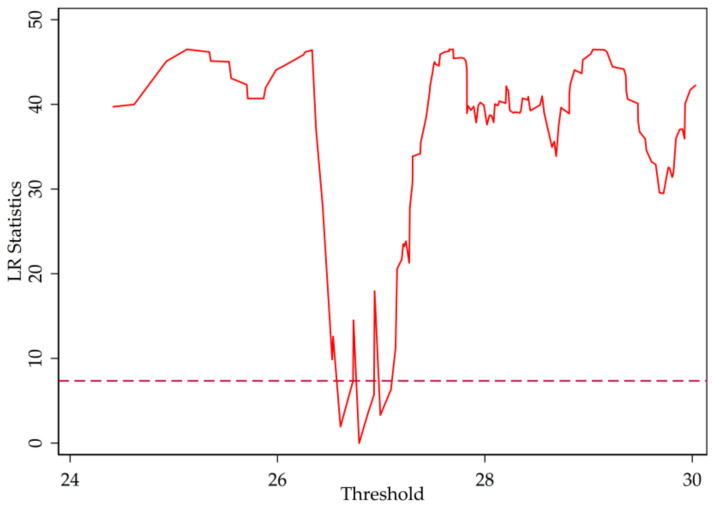
LR statistics (Z-Score).

We followed the research of [55,56,57] and measured the bank size by the total loans of a bank for the robustness analysis of the results. The related threshold tests of models in which the diversification is measured by HHI are listed in Table A1, Table A2 and Table A3. We can conclude that the threshold effects do exist in the models and that there is only one threshold in different models, significant at the 5% level. This accords with the above section and implies that our models are robust. Similarly, the LR statistics of the single threshold of different models (the risk-adjusted return on assets (SHROA), the non-performing loan ratio (NPLR), and the Z-Score are respectively chosen as the dependent variable of the models) are shown in Figure A1, Figure A2 and Figure A3. We also conducted the threshold tests of models in which the diversification is measured by ENTI (see Table A4, Table A5 and Table A6), and derived the same conclusions, besides, the LR statistics of these models are shown in Figure A4, Figure A5 and Figure A6. Note that the blue line represents the critical value at the 95% confidence level of the robust models. 

We derived the threshold parameters by calculation and divide the sample into two groups: Group A (small), 0 < bank size ≤ threshold parameter; Group B (large), threshold parameter ≤ bank size. We find that, when the bank size is measured by the total loans of a bank, for the models of SHROA, NPLR, and Z-Score, 99.31%, 99.31%, and 96.53% of the observations, respectively, belong to the same group as before, which confirms the robustness of our models. 

As demonstrated in Table 12, In Model 7, for small banks, whose size is lower than the threshold parameter, namely 8.2234422 trillion RMB, the rise of diversification leads to the decrease of the bank’s profitability. Meanwhile, for large banks, whose size exceeds this threshold, diversification increases profitability. In Model 8, the result shows that the effect of diversification on the non-performing loan ratio will change from positive to negative if the bank size exceeds the threshold (6.3759167), indicating that large banks are faced with decreased credit risk with the increase in diversification, while small banks tend to increase credit risk. The estimation result of Model 9 shows that diversification plays a significant role in the increase in the solvency risk of a small bank, while the solvency risk of a large bank decreases with the increase of diversification, which shows that large banks are more stable than small banks. This is because a large bank is better known and has a tremendous amount of capital. An increasing number of people prefer to save money in large banks. When large banks carry out income diversification strategies, they make greater profits than do small banks, and their risk decreases as income diversification increases. The robustness test we designed efficiently suggests that the “Matthew Effect” exists in the Chinese banking industry, and large banks perform better than small banks. We can come to the same conclusion based on the estimation results of Models 10, 11, and 12. Additionally, the signs (positive or negative) of the coefficients of all controlled variables are the same as that in the prior models, and most coefficients are significant at the same level. Moreover, some of them even become more statistically significant, for instance, the positive effect of the loan-to-deposit ratio on the overall solvency risk is significant at the 1% level in Model 9, while the effect is significant at the 5% level in Model 3. In all, the above results show that our models are robust.

## 4. Discussion

By using the panel threshold model, we analyzed the relationship between income diversification and a bank’s profitability and risk based on panel data of listed A-share commercial banks from 2008 to 2016. A summary and suggestions for the results are shown below.

According to the calculation in Section 3, we found that the threshold parameter of Model 1 is 10.33739 trillion RMB, which indicates that the size of the “Big Four” Chinese banks (ICBC, CCB, ABC, and BOC) exceeds the threshold parameter, and they are likely to earn more profit by diversification. The threshold parameter calculated for Model 2 is 8.403169 trillion RMB, which shows that the “Big Four” tend to realize a low level of credit risk with diversification. Additionally, the estimation of Model 3 shows a threshold parameter of 1.063901 trillion RMB, and there are 14 banks in the group whose size is greater than this parameter, while the sizes of two banks, NJCB and NBCB, are smaller, which means that, with increased diversification, their solvency risk as measured by Z-score will increase.

Based on the results, our suggestions are as follows: First, large banks tend to earn more profit and have less credit risk and solvency risk; this may be due to superior risk management techniques. Besides, large banks have a long history and thus have accumulated customer resources and formed brand recognition. Additionally, large banks are capital-abundant, while small banks lack the advantage in terms of customer relationships and capital. Meanwhile, there is room to develop diversification of small banks. With the development of Fin-Tech, artificial intelligence techniques such as Deep Learning, and the application of Big Data, the traditional business and non-interest income business of banks have been gradually influenced, so small banks may grow rapidly with diversification if they operate prudently and control risk efficiently. Second, diversification currently benefits large banks; however, if they do not prevent risk efficiently, risk will be system-wide and result in considerable costs. Therefore, regulators should strengthen the supervision of banks, for example, by the use of macro-prudential measures.

Our major contributions are as follows: First, we deepen prior research by analyzing the effect of income diversification on a bank’s profitability and risk. Moreover, we study the effect of diversification on credit risk as measured by the non-performing loan ratio and insolvency risk as measured by the Z-score (the lower the Z-score is, the higher the bank’s financial stability will be). Second, studies on the effect of income diversification have not come to unanimous conclusions; we built a panel threshold model for analyzing the effect of diversification. Diversification was first measured by the Herfindahl–Hirschman index (HHI), and the results show that there is a nonlinear relationship between diversification and profitability or risk. We introduced an interesting index based on entropy to test the robustness of our model and found that the threshold effect exists in both of our models, which is statistically significant. We believe that the combination of the entropy index (ENTI) and the HHI can be used to study the relationship between diversification and profitability or risk more efficiently. Bankers and their customers are becoming increasingly interested in income diversification, and they value risk as well. We suggest that banks of different sizes should adopt the corresponding diversification strategy to achieve sustainable development. In addition, we hope that the combination of ENTI and HHI can be widely used in further studies, and banks can control risk and earn more profit in the future.

## Figures and Tables

**Table 1:** Names and abbreviations of listed banks.

ID	Abbreviation	Name
1	BJCB	Bank of Beijing Co., Ltd.
2	ICBC	Industrial and Commercial Bank of China Limited
3	CEB	China Everbright Bank Co., Ltd.
4	HXB	Hua Xia Bank Co., Ltd.
5	CCB	China Construction Bank Corp.
6	BOCOM	Bank of Communications Co., Ltd.
7	CMBC	China Minsheng Banking Corp., Ltd.
8	NJCB	Bank of Nanjing Co., Ltd.
9	NBCB	Bank of Ningbo Co., Ltd.
10	ABC	Agricultural Bank of China Limited
11	PAB	Ping An Bank Co., Ltd.
12	SPDB	Shanghai Pudong Development Bank Co., Ltd.
13	IB	Industrial Bank Co., Ltd.
14	CMB	China Merchants Bank Co., Ltd.
15	BOC	Bank of China Limited
16	CITICB	China CITIC Bank Corp., Ltd.

**Table 2:** Descriptive statistics of dependent variables.

	Risk-Adjusted ROA (SHROA)	Non-Performing Loan Ratio (NPLR)	Z-Score
Mean	7.447	1.165%	0.023
Min	0.570	0.380%	0.009
Median	6.913	1.040%	0.021
Max	15.278	4.320%	0.063
Standard Deviation	3.218	0.541%	0.010
Coefficient of Variation	0.432	0.464	0.453

Note: ROA is the abbreviation of return on assets.

**Table 3:** The descriptive statistics of independent and controlled variables.

	Diversification (DIV)	Diversification (DEV)	Total Asset (in Million RMB) (ast)	Equity Ratio (eta)	Loan-to-Deposit Ratio (ltd)
Mean	0.308	0.482	5,382,917.772	6.140%	71.068%
Min	0.131	0.254	93,706.071	3.185%	47.426%
Median	0.309	0.487	2,874,202.500	6.179%	72.726%
Max	0.476	0.669	24,137,265.000	12.108%	92.032%
Standard Deviation	0.085	0.101	5,815,194.932	1.201%	8.027%
Coefficient of Variation	0.276	0.209	1.080	0.196	0.113

**Table 4:** Threshold effect test (SHROA).

Threshold	F-Statistics	*p*-Value	1%	5%	10%
Single	22.680	0.030	27.274	18.830	15.991
Double	15.310	0.090	23.594	18.235	14.794
Triple	14.310	0.907	54.147	47.326	41.136

**Table 5:** Threshold effect test (NPLR).

Threshold	F-Statistics	*p*-Value	1%	5%	10%
Single	64.160	0.000	45.516	32.133	27.349
Double	4.610	0.997	54.804	39.428	30.273
Triple	11.360	0.580	61.759	26.329	22.333

**Table 6:** Threshold effect test (Z-score).

Threshold	F-Statistics	*p*-Value	1%	5%	10%
Single	39.330	0.037	67.276	37.474	29.798
Double	17.150	0.290	49.195	37.121	28.113
Triple	14.960	0.373	46.334	33.935	26.897

**Table 7:** Impact of income diversification (DIV) on SHROA, NPLR, and Z-score.

	Model 1	Model 2	Model 3
	SHROA	NPLR	Z-Score
ast	−0.104	−0.002 **	−0.001
	(−0.51)	(−2.24)	(−1.59)
eta	56.226 ***	0.002	−0.402 ***
	(7.13)	(0.05)	(−15.95)
ltd	−6.652 ***	0.010	0.011 **
	(−4.48)	(1.46)	(2.51)
DIV_1	−2.947 *	0.035 ***	0.023 ***
	(−1.75)	(4.46)	(3.92)
DIV_2	1.419	−0.013	0.003
	(0.68)	(−1.29)	(0.54)
constant	12.303 **	0.059 **	0.070 ***
	(2.10)	(2.19)	(3.55)
R^2^	0.412	0.411	0.784
N	144	144	144

Note: ***, **, and * indicate statistical significance at the 1%, 5%, and 10% levels, respectively.

**Table 8:** Threshold effect test (SHROA).

Threshold	F-Statistics	*p*-Value	1%	5%	10%
Single	24.550	0.020	26.400	18.514	16.120
Double	15.820	0.080	22.497	17.295	14.394
Triple	13.530	0.887	54.586	47.995	42.266

**Table 9:** Threshold effect test (NPLR).

Threshold	F-Statistics	*p*-Value	1%	5%	10%
Single	66.250	0.003	48.068	31.469	28.537
Double	7.670	0.923	58.554	40.341	32.687
Triple	12.720	0.480	45.004	25.010	20.487

**Table 10:** Threshold effect test (Z-score).

Threshold	F-Statistics	*p*-Value	1%	5%	10%
Single	41.630	0.030	67.338	37.285	30.089
Double	11.250	0.560	60.883	34.293	27.727
Triple	15.550	0.263	34.637	26.876	22.694

**Table 11:** The impact of income diversification (DEV) on SHROA, NPLR and Z-Score.

	Model 4	Model 5	Model 6
	SHROA	NPLR	Z-Score
ast	−0.167	−0.002 *	−0.001
	(−0.82)	(−1.88)	(−1.47)
eta	55.041 ***	0.007	−0.400 ***
	(6.99)	(0.20)	(−15.99)
ltd	−6.840 ***	0.011	0.011 **
	(−4.62)	(1.51)	(2.39)
DEV_1	−1.912	0.026 ***	0.015 ***
	(−1.36)	(3.97)	(3.26)
DEV_2	1.110	−0.007	0.003
	(0.67)	(−0.89)	(0.58)
constant	14.307 **	0.047 *	0.067 ***
	(2.52)	(1.77)	(3.53)
R^2^	0.411	0.401	0.785
N	144	144	144

Note: ***, **, and * indicate statistical significance at the 1%, 5%, and 10% levels, respectively.

**Table 12:** The impact of income diversification (DIV & DEV) on SHROA, NPLR, and Z-Score.

DIV ^1^	Model 7	Model 8	Model 9	DEV ^2^	Model 10	Model 11	Model 12
	SHROA	NPLR	Z-score		SHROA	NPLR	Z-score
Size ^3^	−0.081	−0.003 **	−0.001 *	size	−0.153	−0.002 *	−0.001 *
	(−0.34)	(−2.35)	(−1.88)		(−0.65)	(−1.98)	(−1.75)
eta	56.463 ***	0.027	−0.386 ***	eta	55.309 ***	0.034	−0.386 ***
	(7.19)	(0.75)	(−16.20)		(7.05)	(0.94)	(−16.33)
ltd	−6.756 ***	0.013 *	0.017 ***	ltd	−6.914 ***	0.013 *	0.017 ***
	(−4.56)	(1.96)	(4.23)		(−4.68)	(1.96)	(4.11)
DIV_1	−3.095 *	0.032 ***	0.026 ***	DEV_1	−2.041	0.024 ***	0.016 ***
	(−1.88)	(4.33)	(4.82)		(−1.48)	(3.78)	(3.76)
DIV_2	1.144	−0.014	−0.003	DEV_2	0.915	−0.008	−0.002
	(0.54)	(−1.32)	(−0.58)		(0.55)	(−0.95)	(−0.52)
constant	11.705 *	0.065 **	0.072 ***	constant	13.919 **	0.052 *	0.069 ***
	(1.83)	(2.24)	(3.71)		(2.23)	(1.82)	(3.69)
R^2^	0.412	0.445	0.809	R^2^	0.410	0.433	0.809
N	144	144	144	N	144	144	144

Notes: ^1^ ***, **, and * indicate statistical significance at the 1%, 5%, and 10% levels, respectively. In Models 7–9, the diversification index is measured based on the Herfindahl–Hirschman index (HHI). ^2^ In Models 10–12, the diversification index is measured by entropy index (ENTI). ^3^ Note that we use the total loans as a measure of the bank size based on the prior literature for robustness analysis.

## Appendix A

**Table A1:** Threshold effect test (SHROA).

Threshold	F-Statistics	*p*-Value	1%	5%	10%
Single	21.570	0.030	25.528	18.121	16.434
Double	13.730	0.197	26.047	20.070	16.144
Triple	8.790	0.800	52.522	43.113	34.683

**Table A2:** Threshold effect test (NPLR).

Threshold	F-Statistics	*p*-Value	1%	5%	10%
Single	77.250	0.000	50.235	37.422	28.938
Double	12.940	0.500	42.831	31.355	25.544
Triple	10.570	0.827	43.552	35.421	30.727

**Table A3:** Threshold effect test (Z-score).

Threshold	F-Statistics	*p*-Value	1%	5%	10%
Single	47.010	0.020	50.568	36.600	28.933
Double	9.790	0.683	53.310	33.234	29.717
Triple	7.740	0.687	38.229	25.427	21.008

**Table A4:** Threshold effect test (SHROA).

Threshold	F-Statistics	*p*-Value	1%	5%	10%
Single	23.650	0.020	26.037	18.372	16.059
Double	12.080	0.257	23.760	18.998	15.574
Triple	9.000	0.770	51.766	41.521	32.870

**Table A5:** Threshold effect test (NPLR).

Threshold	F-Statistics	*p*-Value	1%	5%	10%
Single	81.540	0.000	48.225	35.967	28.572
Double	10.110	0.713	41.873	30.290	25.868
Triple	11.130	0.803	43.836	36.736	31.723

**Table A6:** Threshold effect test (Z-score).

Threshold	F-Statistics	*p*-Value	1%	5%	10%
Single	48.290	0.017	54.529	35.155	30.687
Double	12.360	0.543	50.186	32.922	27.133
Triple	9.680	0.623	64.750	37.673	28.911
